# Elevated blood pressure among primary school children in Dar es salaam, Tanzania: prevalence and risk factors

**DOI:** 10.1186/s12887-018-1052-8

**Published:** 2018-02-13

**Authors:** Alfa J. Muhihi, Marina A. Njelekela, Rose N. M. Mpembeni, Bikolimana G. Muhihi, Amani Anaeli, Omary Chillo, Sulende Kubhoja, Benjamin Lujani, Mwanamkuu Maghembe, Davis Ngarashi

**Affiliations:** 1 Management and Development for Health, Mikocheni, Dar es Salaam, Tanzania; 2 Department of Physiology, Muhimbili University of Health and Allied Sciences, Dar es Salaam, Tanzania; 3 Department of Epidemiology and Biostatistics, Muhimbili University of Health and Allied Sciences, Dar es Salaam, Tanzania; 4 Department of Community and Rural Development, Moshi Cooperative University, Kilimanjaro, Tanzania; 5 Department of Development Studies, Muhimbili University of Health and Allied Sciences, Dar es Salaam, Tanzania; 6 Department of Pediatrics and Child Health, Muhimbili University of Health and Allied Sciences, Dar es Salaam, Tanzania

**Keywords:** Prevalence, Elevated blood pressure, Age, Overweight, Obesity, Children, Tanzania

## Abstract

**Background:**

Whilst the burden of non-communicable diseases is increasing in developing countries, little data is available on blood pressure among Tanzanian children. This study aimed at determining the blood pressure profiles and risk factors associated with elevated blood pressure among primary school children in Dar es Salaam, Tanzania.

**Methods:**

We conducted a cross sectional survey among 446 children aged 6–17 years from 9 randomly selected primary schools in Dar es Salaam. We measured blood pressure using a standardized digital blood pressure measuring machine (Omron Digital HEM-907, Tokyo, Japan). We used an average of the three blood pressure readings for analysis. Elevated blood pressure was defined as average systolic or diastolic blood pressure ≥ 90th percentile for age, gender and height.

**Results:**

The proportion of children with elevated blood pressure was 15.2% (pre-hypertension 4.4% and hypertension 10.8%). No significant gender differences were observed in the prevalence of elevated BP. Increasing age and overweight/obese children were significantly associated with elevated BP (*p* = 0.0029 and *p* < 0.0001) respectively. Similar associations were observed for age and overweight/obesity with hypertension. (*p* = 0.0506 and *p* < 0.0001) respectively. In multivariate analysis, age above 10 years (adjusted RR = 3.63, 95% CI = 1.03–7.82) was significantly and independently associated with elevated BP in this population of school age children.

**Conclusions:**

We observed a higher proportion of elevated BP in this population of school age children. Older age and overweight/obesity were associated with elevated BP. Assessment of BP and BMI should be incorporated in school health program in Tanzania to identify those at risk so that appropriate interventions can be instituted before development of associated complications.

## Background

Elevated blood pressure (BP) in childhood has increasingly become a public health problem of global concern [1]. The prevalence of hypertension in children and adolescents in developing countries has been established through systematic reviews to be between 1 and 5% [2, 3]. In developing countries, particularly African countries, there is a wide variation in the estimated prevalence of hypertension in children and adolescent population ranging from < 5% to as high as > 20% [4–6]. Elevated BP tends to develop during the first two decades of life [7] and may persist through adult life [8]. Risk factors for elevated BP during childhood include age, gender, body size, socioeconomic status, obesity, family history of hypertension, changes in dietary habits, sedentary lifestyle and increasing stress [9–11].

Several studies have demonstrated increasing mean BP with increasing age in children [12, 13]. Obesity is the main determinant of BP in children and adolescents [14, 15]. Blood pressure studies in children provide crucial epidemiological information helpful in the modification of risk factors for coronary heart diseases and other non-communicable diseases later in life [16]. Several studies have consistently reported that elevated BP is significantly correlated with body mass index [17–19]. Identification and modification of risk factors may reduce the incidence and complications associated with elevated BP later in adult life. The 1996 task force report on BP in children recommended that BP measurements be incorporated into routine pediatric examination for children aged 3 years and above [20]. However, such recommendations are rarely followed, mainly because hypertension in the pediatric population is not appreciated and given emphasis like in adults. Despite the greater potential for success rate of lifestyle modification interventions in children as opposed to adults, most prevention and control strategies for hypertension target adult population [21]. Interventions targeting children should be a higher priority for prevention of hypertension and other lifestyle related non-communicable diseases.

The prevalence of hypertension among Tanzania children and adolescents remains largely unknown despite evidence of increasing childhood obesity [22]. It is imperative to understand the prevalence and risk factors for elevated BP in children and adolescents in Tanzania, since there is a greater variability in the reported prevalence from other African countries. Understanding the magnitude and factors for elevated BP in Tanzanian children and adolescents will help in the planning and implementation of appropriate prevention strategies. This study was therefore conducted to estimate the proportion of elevated BP and its associated risk factors among primary school age children in Dar es Salaam, Tanzania.

## Methods

### Study design and participants

We conducted this cross-sectional survey among primary school children aged 6–17 years from 9 randomly selected primary schools in in Dar es Salaam region. Dar es Salaam is the most populated city in Tanzania, with little known information about elevated blood pressure especially among primary school age children. We used a cross-sectional design so as to capture the required multiple data from the selected study population at one point in time.

### Sample size and sampling technique

We determined sample size for the study using EPI INFOR6 STAT CALC based on the following assumptions; expected prevalence of 6% estimated from previous study [23]; significance level 95% and a desired precision of 2%. Of the eligible 542, informed consent and assent was obtained for a total of 466, thus providing a response rate of 86%.

A sampling frame comprised of all primary schools, both public and private obtained from the municipal educational officers of Ilala, Kinondoni and Temeke in Dar es Salaam. We selected schools randomly so as to ensure equal representation of schools from both rural and urban settings. For each school, we randomly selected one class out of seven classes (class I – class VII) and all children and their parents/guardian from the selected class were invited to participate into the study. Children with disability or suffering from a serious illness that could impair anthropometric and blood pressure measurements were excluded from the study.

### Data collection procedures

Data collection methods have been described elsewhere [22, 24, 25]. Briefly, data collection was conducted by trained research assistants using data collections tools comprising of both closed and open-ended questions. Research assistants provided information about the aims, nature, study procedures and measurements to parents and/or guardians of the selected children. A written informed consent was then obtained for parents/guardians who agreed their children to participate in the study. Data collected included; age, date of birth, gender, type of school (public/private), grade, height, weight, blood pressure (systolic and diastolic), number of adults and children in the family, maternal education and occupation, and amount of pocket money given to the child to spend at school. All research assistants wore regular clothing during the entire data collection process, anthropometric and blood pressure measurements.

### Anthropometric measurements

Anthropometric measurements were conducted by trained research assistants early in the morning before starting classes. Anthropometric measurements were conducted in specially prepared room at each school. Children were weighed wearing light clothes and with no shoes. Body weight was measured to the nearest 0.1 kg using a self-calibrating precision digital scale (Omron, Japan) and height to the nearest 0.1 cm using a fixed Shorr measuring board (Shorr Productions, Olner, MD). Body weight and height were then converted to metric measurements for calculation of Body Mass Index (BMI) as weight (kg) divided by square of height (m^2^). All measurements were taken while observing standard precautions [26]. Obesity in this population of primary school children was defined based on BMI percentiles for age and gender. Children with BMI at or above 95th percentile for age and gender were considered obese [27].

### Blood pressure measurements and definition of elevated blood pressure

We measured blood pressure using a standardized digital blood pressure measuring machine (Omron Digital HEM-907, Tokyo, Japan). We took three blood pressure readings following at least 5–10 min of rest. Blood pressure was measured on the left upper arm and in a seated position using age appropriate child blood pressure cuffs. We used an average of the three blood pressure readings during analysis. We calculated SBP and DBP percentiles according to age, gender and height in accordance with the 4th report on diagnosis, evaluation and treatment of hypertension in children and adolescents [28]. Blood pressure status was classified according to SBP and/or DBP percentiles as follows;

- Normal blood pressure: Average SBP and/or average DBP <90th percentile
- Pre-hypertension: Average SBP and/or average DBP ≥90th percentile but <95th percentile
- Hypertension: Average SBP and/or average DBP ≥95th percentile

In this study, we defined elevated blood pressure as average SBP and/or average DBP ≥ 90th percentile for age, gender and height (combination of pre-hypertension and hypertension).

### Statistical analysis

Data analysis ranged from descriptive statistics (means, standard deviations and frequencies) to identification of important associations. The proportion of elevated BP (average SBP and/or average DBP ≥ 90th percentile) and that of hypertension (average SBP and/or average DBP ≥ 95th percentile) were computed. Log-binomial regression models were used to generate relative risk estimates to assess factors associated with elevated BP. Factors assessed in univariate and multivariate analyses included child age (≤10 years and > 10 years), gender (boys and girls), body mass index (underweight, normal weight, overweight and obese), place of residence (rural and urban), number of adults in the family (< 6 people and ≥6 people), number of children in the family (< 3 children and ≥3 children) and amount of pocket money given to spend at school (≤500 and > 500 Tanzanian shillings). The 95% confidence intervals were calculated for both unadjusted and adjusted relative risks. All statistical analyses were performed using Statistical Analysis Software (SAS 9.2, Institute Inc., North Carolina, USA). All the significant tests were 2-sided at a *p*-value ≤0.05.

## Results

Descriptive characteristics of children who participated in the study are summarized in Table 1. The mean age was 11.1 ± 2.0 years. Of the 446 primary school age children included in this analysis, 237 (53.1%) were girls and 249 (55.8%) were residing in urban settings of Dar es Salaam. The mean BMI was 16.6 ± 4.0 kg/m^2^, and proportion of overweight and obesity were 9.8% and 5.2% respectively. Body mass index was significantly higher among girls (*p* = 0.012).

**Table 1:** Socio-demographic and anthropometric characteristics of primary school children in Dar es Salaam, Tanzania

Characteristic	Mean ± SD or N (%)
Age (years)	11.1 ± 2.0
Age category	
≤ 10 years	138 (30.9)
> 10 years	308 (69.1)
Gender	
Boys	209 (46.9)
Girls	237 (53.1)
Place of residence	
Rural	197 (44.2)
Urban	249 (55.8)
Body mass (kg)	34.1 ± 11.8
Height (cm)	142.1 ± 13.3
BMI (kg/m^2^)	16.6 ± 4.0
BMI-defined categories	
Underweight	65 (14.6)
Normal	314 (70.4)
Overweight	44 (9.8)
Obese	23 (5.2)
SBP (mmHg)	103.9 ± 10.3
DBP (mmHg)	65.6 ± 8.2
Blood pressure categories	
Normal BP	376 (84.3)
Pre-hypertension	22 (4.9)
Stage 1 hypertension	39 (8.8)
Stage 2 hypertension	9 (2.0)
Elevated blood pressure	
Yes	68 (15.2)
No	398 (84.8)
Hypertension	
Yes	48 (10.8)
No	378 (89.2)
Number of adults in the family (≥18 years)	
Less than 6 adults	187 (41.9)
6 and above adults	259 (58.1)
Number of children in the family (< 18 years)	
Less than 3 children	201 (45.1)
3 and above children	245 (54.9)
Pocket money to spend at school	
≤ 500 Tanzanian shillings	315 (70.6)
> 500 Tanzanian shillings	131 (29.4)

*BMI* Body Mass Index, *DBP* Diastolic Blood Pressure, *SBP* Systolic Blood Pressure, *SD* Standard Deviation

**Fig. 1:**
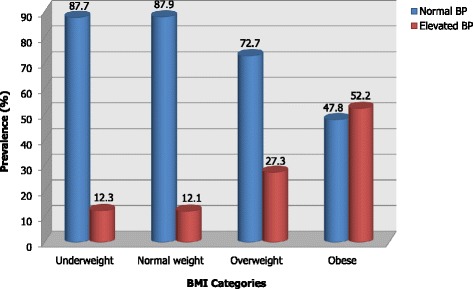
Prevalence of elevated BP and hypertension by BMI category among school age children in Dar es Salaam

Overall, the proportion of children with elevated BP was 15.2% (Pre-hypertension at 4.4% and hypertension at 10.8%). No statistically significant differences were observed in the proportion of elevated BP between boys and girls in this population. Figure 1 summarizes the proportion of elevated BP by BMI categories. Proportion of elevated BP was higher for overweight (27.3%) and obese children (52.2%) compared to 12.3% and 12.1% among underweight and normal weight children. A similar trend was observed for hypertension with 20.4% and 47.8% of overweight and obese children being hypertensive (results not shown).

The associations between elevated BP and other characteristics of the children are shown in Table 2. Children with elevated BP had significantly higher mean values for age, weight, height and BMI (all *p* < 0.001). The mean BMI was 2.7 kg/m^2^ higher for children with elevated BP compared to their counterparts with normal BP. Similarly, prevalence of overweight and obesity was 23% (34.4% vs 11.4%) higher among children with elevated BP compared to those with normal BP. As for age, prevalence of elevated BP was higher among children aged more than 10 years compared to those aged 10 years or less (*p* = 0.0029). Children residing in urban settings of Dar es Salaam had statistically insignificant higher prevalence of elevated BP compared to those from rural settings.

**Table 2:** Socio-demographic and anthropometric characteristics of children with elevated blood pressure in Dar es Salaam, Tanzania

	All	Normal BP	Elevated BP	*p*-value
	Mean ± SD N (%)	Mean ± SD N (%)	Mean ± SD N (%)	
Age (years)	11.1 ± 2.0	11.1 ± 2.1	12.1 ± 1.8	0.0001
Age category				
≤ 10 years	138 (30.9)	127 (33.8)	11 (15.7)	0.0029
> 10 years	308 (69.1)	249 (66.2)	59 (84.3)	
Gender				
Boys	209 (46.9)	174 (46.3)	35 (50.0)	0.5665
Girls	237 (53.1)	202 (53.7)	35 (50.0)	
Body mass (kg)	34.1 ± 11.8	32.5 ± 10.7	42.8 ± 13.7	< 0.0001
Height (cm)	142.1 ± 13.3	140.6 ± 12.8	149.8 ± 13.6	< 0.0001
BMI (kg/m2)	16.6 ± 4.0	16.1 ± 3.6	18.9 ± 5.2	< 0.0001
BMI category				
Underweight	65 (14.6)	57 (15.2)	8 (11.4)	
Normal weight	314 (70.4)	276 (73.4)	38 (54.2)	< 0.0001
Overweight	44 (9.8)	32 (8.5)	12 (17.2)	
Obese	23 (5.2)	11 (2.9)	12 (17.2)	
Place of residence				
Rural	197 (44.2)	169 (44.9)	28 (40.0)	0.4441
Urban	249 (55.8)	207 (55.1)	42 (60.0)	
Number of adults in family				
Less than 6 adults	187 (41.9)	157 (41.8)	30 (42.9)	0.8638
6 and above adults	259 (58.1)	219 (58.2)	40 (57.1)	
Number of children				
Less than 3 children	201 (45.3)	163 (43.4)	38 (54.3)	0.0914
3 and above children	245 (54.7)	223 (56.6)	22 (45.7)	
Pocket money for school				
≤ 500 Tanzanian shillings	315 (70.5)	268 (71.3)	47 (67.1)	0.4857
> 500 Tanzanian shillings	131 (29.5)	108 (28.7)	23 (32.9)	

*BMI* Body Mass Index, *BP* Blood Pressure, *DBP* Diastolic Blood Pressure, *SBP* Systolic Blood Pressure, *SD* Standard Deviation

Unadjusted and adjusted analysis of risk factors for elevated BP is presented in Table 3. Children above 10 years of age had a 3.6 times higher risk for elevated BP compared to their counter parts aged 10 year or less [adjusted RR = 3.63 (95% CI: 1.03–7.82), *p* = 0.0450]. Although not statistically significant, overweight and obese children had increased risk for elevated BP compared to normal weight children [adjusted RR = 1.82 (0.21–5.73)] and [adjusted RR = 2.21 (0.12–3.67). Having 3 or more children was protective against elevated BP with a borderline significance in univariate analysis, but this protective effect disappeared when other factors were taken into consideration in the multivariate analysis.

**Table 3:** Relationship between age, gender, body mass index and sociodemographic factors with prevalence of hypertension among primary school children in Dar es Salaam, Tanzania

Variable	Unadjusted RR (95% CI)	*P* value	Adjusted RR^a^ (95% CI)	*P* value
Age				
≤ 10 years	Ref		Ref	
> 10 years	5.46 (1.60–8.62)	0.0067	3.63 (1.03–7.82)	0.0450
Gender				
Girls	Ref		Ref	
Boys	1.08 (0.70–1.67)	0.7318	1.07 (0.70–1.61)	0.7604
BMI (kg/m^2^)				
Underweight	1.07 (0.52–2.19)		1.22 (0.59–2.51)	
Normal	Ref		Ref	
Overweight	1.21 (0.14–6.46)	0.8602	1.82 (0.21–5.73)	0.5885
Obese	1.29 (0.07–2.87)		2.21 (0.12–3.67)	
Place of residence				
Rural	Ref		Ref	
Urban	1.44 (0.59–3.53)	0.4250	0.99 (0.37–2.61)	0.9795
Number of adults in family				
Less than 6 adults	Ref		Ref	
6 and above adults	0.94 (0.39–2.28)	0.8909	1.13 (0.47–2.74)	0.7817
Number of children				
Less than 3 children	Ref		Ref	
3 and above children	0.43 (0.18–1.03)	0.0581	0.47 (0.19–1.18)	0.1076
Pocket money for school				
≤ 500 Tanzanian shillings	Ref		Ref	
> 500 Tanzanian shillings	1.49 (0.59–3.73)	0.3936	0.98 (0.39–2.47)	0.9673

*RR* Risk Ratio, *BMI* Body Mass Index, *CI* Confidence interval

^a^Risk ratio adjusted for age, gender, place of residence, BMI, number of adults and children in the family, pocket money and BMI category

## Discussion

The current study presents the findings of blood pressure profile among primary school age children from rural and urban settings of Dar es Salaam, in Tanzania. Our findings demonstrate a higher proportion of elevated BP of 15.2% and that of hypertension (stage I and stage II) of 10.8% in this surveyed population of primary school children. Blood pressure increased with age in both boys and girls.

Our finding of elevated BP are comparable to other studies conducted in Tanzania [23, 29] . However, there are some differences in the definition of hypertension worth noting. Contrast to our study which considered hypertension as having either systolic and/or diastolic hypertension as per the fourth report on the diagnosis, evaluation and treatment of high blood pressure in children and adolescents [28], both studies by Chillo et al. and Mushengezi et al. [23, 29] reported the prevalence of isolated SBP and DBP and that of combined. Our study therefore provides a good estimate of the prevalence of both pre-hypertension and hypertension in this population of school age children in Dar es Salaam.

The prevalence of elevated BP among children is showing a declining trend in some countries like United States [30] Seychelles [31] and Japan [32], however, other countries such as United Kingdom [33] and Peoples Republic of China [34] have reported increasing trend. Studies conducted elsewhere in Africa among children and adolescents have also reported elevated blood pressure levels [35, 36]. The proportion of children with elevated BP reported in our study is also alarming and factors associated with the increase need to be explored and addressed urgently.

We did not observe statistically significant association between overweight/obesity with elevated BP. However, overweight and obese children had 1.8 and 2.2 times higher risk for elevated BP respectively compared to normal weight children. Studies conducted elsewhere in Africa have reported association of overweight/obesity with elevated BP. A study conducted among urban South African children aged 6 to 13 years found higher BP among children with higher BMI [37]. Similar relationship between BMI and BP has also been reported in Ghana and Cameroon studies [5, 38].

Our findings of higher prevalence of elevated blood pressure in older children concur with other studies conducted in Africa [39–41]. Age-related increase in blood pressure is partly attributable to increasing weight with age. The correlation between BP and BMI in children highlights the development of metabolic syndrome, a relationship that has already been established [42]. However, our findings contrast with those reported by Chiolero et al. [31], who found higher prevalence of elevated BP among younger children. Their possible explanation for such findings were a reaction alert (white coat hypertension) among younger than older children and a measurement bias due to use of automated blood pressure device (larger versus smaller arms). Although we also used automated blood pressure measuring devices in our study, we used age appropriate blood pressure cuff size to help minimize measurement bias.

Our findings of lack of gender difference in the prevalence of elevated blood pressure concur with reports by Chillo et al. and Mushengezi et al. [23, 29]. Male gender is however a well known risk factor for elevated BP in children and adolescents [43]. Sex steroids have been strongly implicated in explaining the differences in risk of high blood pressure [44]. Lifestyle related behavior such as sedentary lifestyle / physical inactivity could account for the gender differences in the prevalence of elevated BP in children. Studies on physical activity have indicated gender differences in the level of physical activity and that boys are likely to be more physically active than girls [45]. Physical activity may have a blood pressure-lowering effect through increased capillary formation [46]. However, this study did not assess physical activity in the studied population of school age children and cannot draw any conclusions on the association between physical activity and prevalence of elevated BP.

Our analysis has several limitations. The sampled children may not represent the children in Dar es Salaam or Tanzania. Therefore, the generalizability of these finding to the greater Tanzanian children must be done with caution. Although we took three blood pressure measurements, 5–10 min apart, they were all taken on one occasion, contrary to the recommendations [28], that blood pressure measurements be taken on three different occasions or using ambulatory blood pressure monitoring. This method may lead to elevated blood pressure in this population being overestimated due to white coat hypertension in children. It has been shown that prevalence of elevated blood pressure may be decreased by about half on repeated blood pressure measurements [47]. Due to short time of the study and funding limitations, we did not assess many several other factors such as birth weight, dietary information, physical inactivity and genetic factors that may have affected our findings.

## Conclusion

High blood pressure during childhood and adolescent period is a strong risk factor for development of cardiovascular diseases later in adulthood [48, 49]. Based on the methodology we used, the proportion of children with elevated BP was 15.2% (4.4 pre-hypertension and 10.8% hypertension. Without undermining the need for conducting other studies in children and adolescent population that will BP measurements on different occasions or ambulatory BP monitoring, to better estimate the magnitude of hypertension, the findings this study highlight the need for immediate interventions in primary schools including incorporation of assessment of BP and BMI in school health programs in Tanzania. Such school health programs would enable identification of children with elevated BP and BMI at an early stage for appropriate interventions to be instituted, hence prevent development of serious health complication during childhood and later in adult life. Further studies should also assess and fully explore factors associated with elevated BP in children in resource limited settings like Tanzania.

## Abbreviations

BMI: Body mass index
BP: Blood pressure
DBP: Diastolic blood pressure
RR: Relative risk
SAS: Statistical analysis software
SBP: Systolic blood pressure
